# Microvesicles in CLL: predictor of disease progression/relapse

**DOI:** 10.18632/oncotarget.21868

**Published:** 2017-10-17

**Authors:** Guru P. Maiti, Neil E. Kay, Asish K. Ghosh

**Affiliations:** Asish K. Ghosh: Stephenson Cancer Center and Department of Pathology, The University of Oklahoma Health Sciences Center, Oklahoma City, OK, USA

**Keywords:** microvesicle, exosome, CLL, biomarker

Tumor-secreted extracellular vesicles (EVs) including microvesicles (MVs) and exosomes, classified according to their sub-cellular origin [1], are critical mediators of intercellular communication between malignant cells and cells in local and distant microenvironments supporting metastatic niche formation. Clinically, EVs may be biomarkers and novel therapeutic targets that reflect cancer progression, and in particular for predicting and preventing metastatic development [2]. Different biological circumstances under which MV formation has been observed reflect the diversity of their biogenesis. Thus, cellular activation, transformation, stress or programmed cell death are associated with a varying MV production output and their biologic features [3]. Previously, we identified increased plasma levels of predominantly platelet/megakaryocyte-derived MVs in early stage chronic lymphocytic leukemia (CLL) and also documented a phenotypic shift towards more leukemic B-cell-derived MVs with advanced disease stages [4].

Most recently, we studied dynamics of MV generation *in vitro* by CLL B-cells and *in vivo* in CLL patients, and their potential use as predictor for CLL progression, therapeutic response and relapse [5]. We find that while CLL B-cells are capable of generating MVs *in vitro*, CLL B-cells with unmutated IGVH produce higher levels of MVs with B-cell receptor (BCR)-ligation. Further analysis suggests that the tonic BCR signal in CLL B-cells may not be the only factor influencing spontaneous shedding of MVs, as targeting the BCR signal could not block MV production from CLL B-cells, whereas it is known to block exosome production [6]. However, the reduction of CLL plasma MVs following BCR-targeted therapy reinforces the possibility that plasma MV levels can be biomarkers for CLL disease status. Of relevance, MV production from CLL B-Cells is likely to be complicated involving multiple intrinsic and/or tumor microenvironmental factors.

Depending on the cell type, membrane MVs may be rich in cell-specific lineage markers [7, 8]. Interestingly, CLL B-cells generate primarily CD52^+^ MVs, but not the B-cell lineage-specific marker CD19^+^ MVs, as we have found *in vitro* with CLL B-cells. This latter result was similar with or without *in vitro* BCR stimulation and importantly, MVs from CLL plasma. In partial explanation we found that both normal B- and CLL B-cells express significantly higher levels of CD52 than CD19 while the CD52 expression levels on normal B-cells were about threefold higher than CLL B-cells. Given this, we hypothesized that lower levels of CD52 on CLL B-cells are a result of shedding primarily CD52^+^ MVs. Indeed, expression levels of CD52, but not CD19, on CLL B-cells were reduced significantly following 72-hour culture accompanied by increased production of CD52^+^ MVs. Further analysis suggests that shedding of CD52^+^ MVs is a physiologic characteristic of human B-lymphocytes. Consistent with the above observations, an increase of plasma CD52^+^ MV level was noted in 7 of 9 follow-up untreated CLL patients as they progressed from a more indolent phase; while no significant alteration of CD52^+^ MV levels was discernible in CLL patients with stable disease. These early findings indicate that plasma levels of CD52^+^ MVs can be a progression biomarker but do need further validation in much larger CLL cohorts.

To further our knowledge of *in vivo* MV dynamics, we studied MV parameters in plasma from a pre- and post-therapy cohort of 33 CLL patients. Although plasma CD52^+^ MV levels dropped in the majority of CLL patients 6-12 months after therapy, in ∼48% cases, CD52^+^ MV levels rose over time with no apparent sign of clinical progression. The observed rise of post-therapy plasma CD52^+^ MV levels appeared to be independent of the status of the clinical response to therapy. Two relevant clinical observations were: (i) elevation of CD52^+^ MV levels in post-therapy CLL plasma at early time points with clinical manifestation of the disease that was only noted at a later time point (*n* = 10) where patients also required subsequent treatment; and (ii) elevation of CD52^+^ MV levels in post-therapy CLL plasma (*n* = 16) where there were no clear signs of clinical progression. Although intermittent elevations of CD52^+^ MV levels can be a result of leukemic B-cell activation in patients who have infections or inflammatory complications, a steady and consistent rise of CD52^+^ MV levels we believe is most likely an early indication of leukemic growth and future relapse. Further studies are needed to validate this possibility as it does hold the promise of predicting occurrence of relapse earlier than traditional clinical manifestations of the disease.

**Figure 1:**
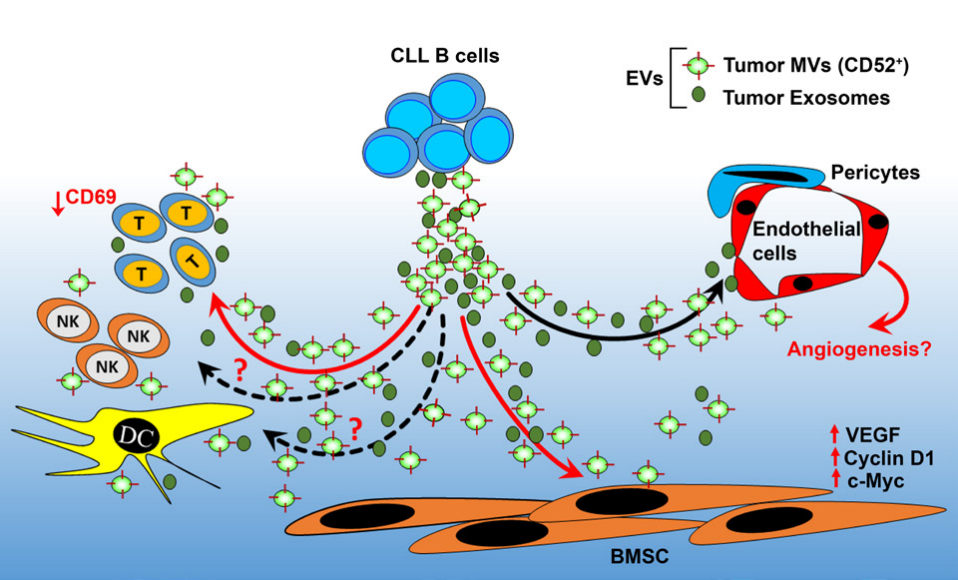
Role of circulating EVs in reprograming CLL tumor microenvironment Increasing number of studies indicate that circulating EVs may play a critical role in modulating CLL tumor microenviroment. CLL MVs are shown to activate the bone marrow stromal cells (BMSCs) via transfer of bioactive molecules including Axl receptor tyrosine kinase. In turn, the activated BMSCs produce increased levels of VEGF, a survival factor for the leukemic B-cells. Interaction of CLL BMSCs with CLL MVs has also been shown to activate the β-catenin signaling pathway leading to increased expression of cyclin D1 and c-Myc, important regulators of cell cycle. On the other hand, CLL-derived exosomes are actively incorporated by endothelial cells and mesenchymal stem cells ex vivo and in vivo and that the transfer of exosomal protein and microRNA induces an inflammatory phenotype in the target cells, which resembles the phenotype of cancer-associated fibroblasts. CLL-EVs released by activated CLL B-cells are reported to augment immune synapse activity while downregulating CD69 expression levels on CD4^+^ T cells. In total it is likely that CLL EVs target diverse cell types in bone marrow or other lymphoid tissues to reprogram the function of CLL tumor microenvironment favoring the disease process.

Tumor cell-derived EVs carry a diverse biologic cargo and are able to reprogram the behavior of the target cells at distant tissues or at close proximity likely promoting the development of an environment hospitable toward cancer growth, invasion and metastasis. Thus, apart from the potential value of circulating EVs in diagnosis or as predictor of therapeutic response in cancer, targeted inhibition of EV production may impair/delay the metastasis process. EVs released from CLL B-cells may also target and alter the functions of various tissue cell types including immune cells, endothelial cells or bone marrow stromal cells (Figure 1). These interactions in turn may lead to other clinical complications in CLL such as impaired immune response of the CLL patients to infection. Decoding how cancer-derived EVs mediate intercellular communication and harnessing these mechanisms for diagnostic and therapeutic purposes holds unique promises to further understand cancer’s systemic effects and to improve the standard of care for cancer patients.
